# Improving access to palliative care for patients with cancer in Africa: 25 years of Hospice Africa

**DOI:** 10.3332/ecancer.2019.946

**Published:** 2019-07-25

**Authors:** Anne Merriman, Eddie Mwebesa, Ludo Zirimenya

**Affiliations:** 1 Hospice Africa Uganda, PO Box 7757, Kampala, Uganda; 2 Medical Research Council/Uganda Virus Research Institute and London School of Hygiene and Tropical Medicine Uganda Research Unit, Entebbe

**Keywords:** African palliative care, oral affordable morphine, total suffering

## Abstract

All cancer care must target the needs of the whole population, not just the few who reach curative services. This paper will refer to palliative care in Uganda and in the countries now aware of the need for palliative care. Human Rights Watch has declared that doctors who are aware that we can control cancer pain and are not doing it or taking steps to make it happen, are considered to be torturers (Human Rights Watch (2009) **Please, do not make us suffer any more...** Accessed 11 July 2019).

As Uganda celebrates 25 years since the introduction of palliative care, is it now time to harvest the principles that have been applied in policies and services from the Government of Uganda? This has brought Uganda to the same level as the developed world as stage 4b palliative care (PC) services [1]. These policies and services need to be promoted to caring governments in Africa, and suitably adapted to the needs of each African country, with a plan for them to progress over the next 5 years. These steps will ensure standards, economic viability and cultural appropriateness.

Let palliative care reach at least 50% of cancer patients in need in Africa by 2023.

## WHO definition of palliative care

*Palliative care is an approach that improves the quality of life of patients and their families facing the problems associated with life-threatening illness, through the prevention and relief of suffering by means of early identification and impeccable assessment and treatment of pain and other problems, physical, psychosocial and spiritual* [2].

## Palliative care: a public health problem

In 2007 [3, 4], cancer was declared a public health problem with the emphasis on the scarcity and the expense of chemotherapy and radiotherapy. The majority of the sick are not reaching curative services.

The biggest cause of cancer suffering today, in the majority of African lower middle-income countries (LMICs), is the lack of access to any form of treatment where 97% of cancer patients do not reach curative care. (97%, not reaching cancer therapy, is from Uganda which has one radiotherapy machine and oncology now reaching several sites. 33% of countries do not have a radiotherapy machine (note: This comes into the text below and referenced as ten) and many have no oncologist, although simple chemo is given by physicians) They remain in excruciating pain in rural areas and in urban slums, and the helpless family members suffer also. Who cares about them?

## The need for palliative care in Africa today

Palliative care is necessary for all who are facing a life threatening illness. This includes cancer, AIDS and many other conditions. The growing incidence of non-communicable diseases (NCDs), due to an increasing ageing population in the world and Africa, is drawing attention to this need. This paper is dedicated to the needs for palliative care for cancer patients, which in training others, enables them to deliver palliative care to those with NCDs and other life threatening conditions.

## Policies from Ugandan Government and HAU Education aspects

Recognising the lack of medication for severe pain and limited access, due to the shortage of prescribers, doctors being the only legal prescribers in most countries; the Uganda Government have:

1. Added to the statute allowing midwives to prescribe pethidine for women in labour, so that nurse specialists, trained with a diploma in clinical palliative, can now be legal morphine prescribers since 2003. This has just been endorsed by INCB in their recent report [5].
2. Provided free oral morphine for all in severe pain and prescribed by a legal prescriber, since 2004.
3. Shared a Public Private Partnership (PPP) with Hospice Arica Uganda (HAU) to manufacture oral morphine for the whole country since 2011.
4. Education: PPP in education commenced in 1994 with teaching of undergraduate medical students, so all doctors qualified since then understand PC. This was between Makerere University and HAU, now the Institute for Hospice and Palliative Care in Africa, which trains post graduates and health professionals up to degree level in African palliative care. 10,000+ have been trained since 1993.
5. Education in Africa: the International Programmes Department at HAU has trained 386 African Initiators from 30 countries and visited several of these countries to give training and support. Two 5 week programmes per year are given in Uganda. This includes 2 weeks of teaching and sharing between countries, 2 weeks on mobile rounds and teaching in the homes and the last week is Training of Trainers. There is one in English and one in French, annually. They leave to practice, to teach and to advocate palliative care. They are further supported through their alumni.

## Age matters

In many African countries, the over 65’s make up less than 4% of the population [1], in some countries in the developed world the same age group makes up as much as 23% [6]. But that small percentage of the huge population of Africa still gives large numbers. The elderly do not have the strength to change their situations. Most have no pension, no health coverage and rely on family or the good will of neighbours to assist them. This generation of the elderly have lost many family members to AIDS in the days before anti retro-virals (ARVs) and even now when they are unable to access them. Thus, family support has reduced for them. Also those who originally had HIV and have survived are few. Many of that cohort died before the intervention of ARVs. Thus, in Uganda, the percentage of elderly is falling and now less than 2% [7].

In many countries, children represent the vast majority of the population. In Uganda, 50% of the population is below 15 and this percentage does not fall below 40 in most of the poorer African countries [7]. Childhood cancers, low in percentage, can also be large in numbers. Many cases are not brought forward from rural areas to reach curative services. Furthermore, there is a preference for the boy child in Africa, thus the boys are brought for treatment whilst the girls are left in the community to suffer [8, 9].

Cancer is striking all ages and increases with age. It is increasing proportionately in line with NCDs. Cancer has doubled in countries with a higher incidence of HIV [10]. The curative services are still only available to around 5% of cancer patients. One third of the countries in Africa do not even have one radiotherapy machine [11].

The greatest cancer incidence is found in the age range of 20–50: the child-bearing ages [12]. Much suffering is found, especially in women suffering from cancer of the cervix and breast. This suffering is qualified as ‘total pain’, which includes physical, psychological, social, economic, cultural and spiritual. The role of women and the value of the woman vary in different cultures. If a woman is bought with a bride price, similar to buying a cow, she may be treated as such, and when diseased she will be set aside and neglected. At least the cow can be put down. The suffering in African woman with cancer is something you need to see to believe. In spite of severe physical suffering their greatest worry is ‘what will happen to my children’ [13].

These factors need to be understood in palliative care in Africa, and addressed. They will differ from tribe to tribe and country to country.

## African Palliative Care (APC)

We cannot estimate the need for cancer in Africa without knowing the statistics for cancer. We are using a simple formula [14] for the prevalence of cancer in an African country. This is based on the incidence of cancer in African countries before independence, which was estimated at 0.1%. AIDs and other viral diseases have doubled this incidence to 2% and prevalence will include those cancers surviving from previous years (remembering very few survive cancer when curative treatment is so scarce) adding another 1%. As palliative care is needed from diagnosis of a life limiting illness, it should be commenced immediately but of course many are unknown in communities. The prevalence is 0.3% of the population (for cancer alone). Thus for example: Uganda has a population of 40 + M [6] The expected need of PC among cancer patients is (40M/100)*0.3 = 120,000.

Services should be planned through education and training to meet the needs of this estimate for cancer.

Table 1 [15] shows calculations of need for the nine African countries attending the 2018 Initiators Programme for Francophone countries delivered by the international programmes of HAU. This is estimated by all the participants for their own countries using statistics. These are calculated using the formulas for Cancer and AIDS.

- What is APC?
  African Palliative Care is one that [16]:
  - Can deliver patient and family centred care.
  - Has affordable oral medication to control pain and symptoms in the home.
  - Is affordable and sustainable in the LMIC, as advised by the recent Lancet Commission report 2018 [17] (as low as USD3 per oral morphine pain control for the average patient, at HAU)
  - Is delivered in the place most acceptable to the patient and family (research in five African countries in 2003 showed that both patients and carers preferred to die at home as long as there was a suitable home care service [18]).
  - Uses African solutions to African challenges. There are affordable home care appliances that can be made in the village as long as it is improving the patient’s quality of life and independence.
  - Is understood and carried out at all levels of care. This requires impeccable service, education, advocacy and follow up.
- Principles of APC:
  1. The public health statistics for each country are essential to make an estimate of the need for cancer patients and to plan a service so that ‘no one is left behind’.
  2. Principles arising from our ethos for Africa [19]:
    - Patient and family needs are paramount. All decisions, whether at a patient or a policy level, must be made while understanding the effect this will have on the patient and their family.
      Pain and symptom control is essential so that a holistic approach to care is possible. Oral affordable morphine is the best analgesic for severe pain [20] and the most affordable for use in African homes [21].
    - Teams work together in harmony to bring hospitality to patients and families.
    - Working together with partners, in harmony. Aware that rivalry for funds can disrupt a coordinated service. Palliative care providers will work together with other providers to ensure all are cared for without doubling of services to a few and leaving others behind. Respect, recognition for each other and sharing is essential.
- Working together with service partners:
  1. The government is essential for palliative care to move forward in a country [22]. They should, therefore, be involved from the start, and policies discussed and followed through with the Ministry of Health. Other government ministries are involved with regulations regarding: class A medicines, the recognition of the specialty and the employment of palliative care trained personnel in specialist areas.
  2. Palliative care often commences with one service, preferably as an NGO, free from government or other bureaucratic regulations, where the spirit of autonomy to the end of life for patients is followed [21]. Once the ethos and spirit is embedded in palliative care, this can be transmitted to hospitals in order for patients to be seen as the person and not the disease. PPPs would be the way forward, with governments supplying funding and NGO’s expertise, working together for a comprehensive programme reaching all in need.
  3. A country palliative care association should be envisaged and commenced to coordinate services throughout the country, provide continuing medical education and publications and to support new initiatives in service and training. It also becomes the lead voice to the government for to advocate for growth and policies for palliative care reaching all.
  4. Other palliative care providers are included. New specialist services may arise for different diseases. AIDs support organisations need palliative care. Increasingly NCD teams need to be trained in palliative care also.
- It is essential to adapt to differing cultural beliefs and diversity, spirituality and religious beliefs, language, economy, population statistics and medical services. Thus the principles outlined above will have to be modified to suit the best way to move forward in the knowledge of each situation. These will also be adapted to changes in social norms in each country, with time.
- Finally, it is critical to spread care through maintaining quality education, advocacy and sustainable government integration. The palliative care association in each country will be the coordinating group to carry out government advocacy, coordinate sharing through meetings and publications to support teams throughout the country [23].

## The role of oral morphine in LMICs

Palliative care was first introduced to Africa in Zimbabwe in 1979. South Africa commenced the following year. There was then a 10-year gap until Nairobi Hospice commenced in 1990. The gap was because the LMICs could not afford the medications for the severe pain of cancer. In many countries and places, the strongest analgesic was paracetamol. Despite a significant improvement in palliative care services in LMICs, there is still limited palliative care development [24].

Affordable oral morphine was first introduced to Africa to Nairobi Hospice in 1990 by AM (see authors), who had designed this cheap pure morphine formula for use in home care in Singapore, where she and nurse volunteers commenced their home care service. This has allowed the relief of the severe pain which had blocked holistic care, addressing needs for the future, fears and spiritual distress.

The availability of free morphine to control pain has progressed in Uganda. Oral liquid morphine produced at HAU for the whole country is given free to all who are prescribed it by a registered prescriber [21]. Nurse prescribers have allowed palliative care to be available in 90% of the Districts.

## Education

Education and training is the way to spread palliative care [22]. Efforts should be made in each country to introduce palliative care into the nursing and medical curricula, at undergraduate and postgraduate levels. Clinical experience should be in the communities as well as in hospitals.

In Africa, nurses are the leaders and the main professionals in palliative care. Training of pharmacists and others including those working in communities—community volunteers, family carers, spiritual advisors, traditional healers etc.—is also necessary to provide a comprehensive patient friendly service.

**Figure 1.**
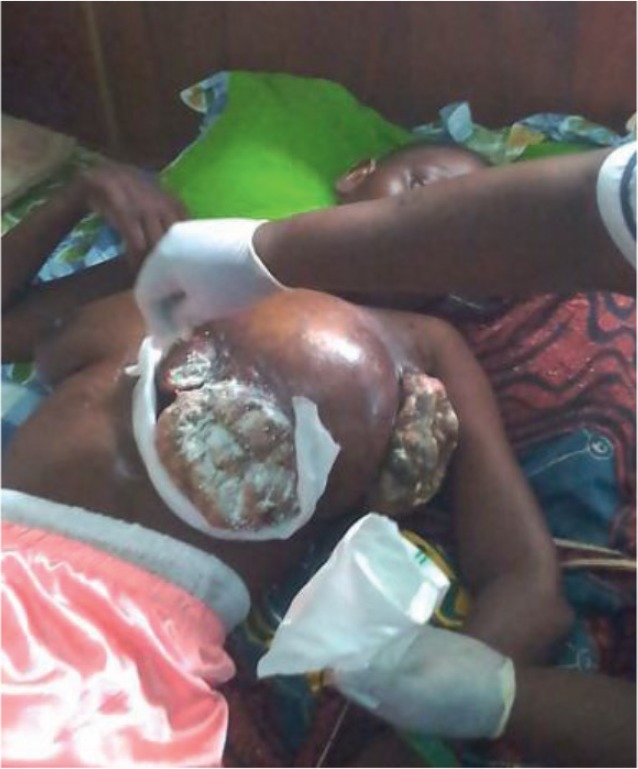
A young lady, found at home in Cameroon without medical help. After going for initial treatment, she did not return, as she could not afford it. (Courtesy of Dr Esther Mbassi Dina Bell.)

**Figure 2.**
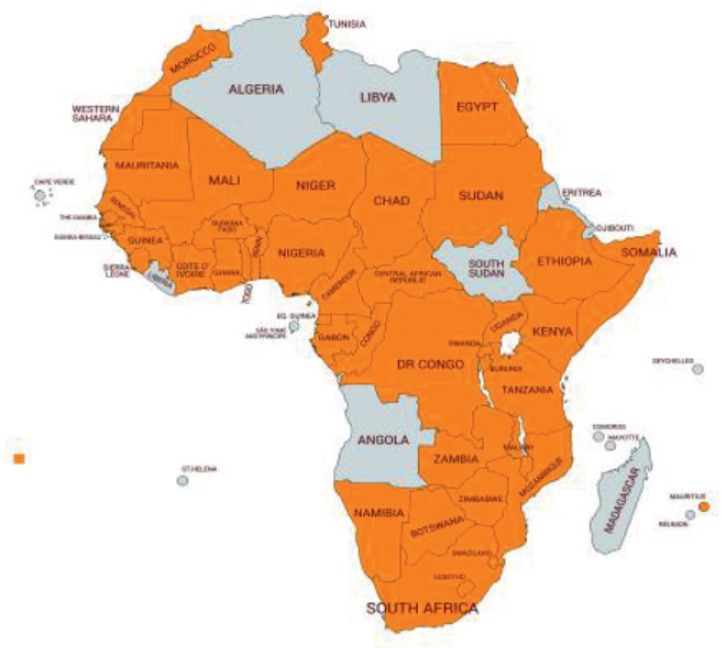
African countries that now know and practice palliative or support care since 1993, when only three countries were practising in Africa. 22 have affordable oral morphine. (Courtesy of Daisy Bartlett.)

Figure 1 shows a case from Cameroon of extending cancer of the breast, which is a recurring issue when follow-up into the home is not available. Figure 2 shows the increase in countries with palliative care from 3 countries in 1993, when Hospice Africa commenced, with a vision of "Palliative care for all in need in Africa", to 35 in 2018.

## Funding

Finding funding is the greatest challenge in Africa today for the bringing of palliative care to all of those in need. Indeed, some African countries are contemplating closure due to financial straits. This is also affecting Uganda, which is already cutting down on patient services and education.

Why is this? In the light of the recession affecting donor funding, they have rearranged their priorities. Governments and donors see palliative care as the lowest priority in health even though we are all faced with our own death and those without treatment the most painful death. Also, several African countries have been blacklisted because of corruption, yet corruption is omnipresent in today’s world. The suffering in Africa is huge.

Donors are still prioritising AIDS. Yet statistics show that if the incidence of HIV is below 6% then the palliative care burden is higher for cancer than AIDS [13]. West and North Africa have the lowest incidence of HIV. Southern Africa still has the highest levels with East Africa following.

It is time for funds to be concentrated on the suffering of the African cancer patient. Dying is everybody’s business. We come into the world naked and go out naked. So, if you have a surplus of money, remember, you cannot take it with you! See the suffering and have the compassion to do something about it.

## Conclusion

Hospice Africa has set the way forward for African palliative care suitable to every country. This progress is in danger of ceasing. This is because of 1) lack of political will, with patient care as the centre of all decisions including importing affordable morphine powder to make up the solution and 2) lack of funding to give sustainability.

Both of these obstacles should be overcome with advocacy and support for the suffering, as well as awareness and treatment of total pain, from our oncologists and other colleagues.

## Finally

Space limits more exhaustive and comprehensive suggestions. Readers are referred to the IPRI publication: **The State of Oncology in Africa 2015,** which gives a more comprehensive report of palliative care in Africa [14].

## Conflicts of interest

The authors have no conflicts of interest to declare.

## Funding

This article was not supported by any funding.

## Figures and Tables

**Table 1.** Number of people in need of palliative care in nine Francophone African countries.

HDI rank 2016	Country	Pop M	% HIV	PC HIV Needs	Cancer PC Needs	Total PC Needs: '000
135	Congo Brazzaville	5	2	5,000	15,000	20
153	Cameroon	25	0.25	3,100	75,000	78.125
157	Mauritania	3.8	0.5	950	12,000	12.95
166	Togo	8	0.08	320	24,000	24,32
167	Benin	11	1	5,500	33,000	38,5
176	DRC	83.3	0.7	29.000	249,000	278
183	Guinea C	13.2	1.7	9,750	39,000	48.75
185	Burkino Faso	20	0.2	2.000	50,000	62
187	Niger	19,2	0.4	3,840	58,000	61.8

HDI: human development index
